# Human iNPC therapy leads to improvement in functional neurologic outcomes in a pig ischemic stroke model

**DOI:** 10.1002/brb3.972

**Published:** 2018-04-18

**Authors:** Vivian W. Lau, Simon R. Platt, Harrison E. Grace, Emily W. Baker, Franklin D. West

**Affiliations:** ^1^ Regenerative Bioscience Center University of Georgia Athens GA USA; ^2^ Department of Animal and Dairy Science University of Georgia Athens GA USA; ^3^ Department of Small Animal Medicine and Surgery University of Georgia Athens GA USA

**Keywords:** functional outcome scale, induced pluripotent stem cell, large animal stroke model, neural progenitor cell, porcine stroke model

## Abstract

**Introduction:**

Stroke is the leading cause of disability in the United States but current therapies are limited with no regenerative potential. Previous translational failures have highlighted the need for large animal models of ischemic stroke and for improved assessments of functional outcomes. The aims of this study were first, to create a post‐stroke functional outcome assessment scale in a porcine model of middle cerebral artery occlusion (MCAO) and second, to use this scale to determine the effect of human‐induced‐pluripotent‐cell‐derived neural progenitor cells (iNPCs) on functional outcome in this large animal stroke model.

**Materials and Methods:**

Eight 6‐month‐old Landrace mix pigs underwent permanent MCAO. Five days following MCAO, pigs received intraparenchymal injections of either iNPCs or PBS. A post‐stroke assessment scale was developed to measure functional outcome. Evaluations were performed at least 1–3 days prior to MCAO and repeated 1 day, 3 days, and 5 days post‐stroke as well as 1 day, 3 days, 1 week, 2 weeks, 4 weeks, 6 weeks, 9 weeks, and 12 weeks post‐injection. Comparisons of scores between animals receiving iNPCs or PBS only were compared using a two‐way ANOVA and a Tukey's post‐hoc *t* test.

**Results:**

The developed scale was able to consistently determine differences between healthy and stroked pigs at all time points. iNPC‐treated pigs showed a significantly faster recovery in their overall scores relative to PBS‐only treated pigs with the parameters of appetite and body posture exhibiting the most improvement in the iNPC‐treated group.

**Conclusions:**

We developed a robust and repeatable functional assessment tool that can reliably detect stroke and recovery, while also showing for the first time that iNPC therapy leads to functional recovery in a translational pig ischemic stroke model. These promising results suggest that iNPCs may 1 day serve as a first in class cell therapeutic for ischemic stroke.

## 1 INTRODUCTION

Stroke is the leading cause of disability in the United States and the second leading cause of death in the world (Mozaffarian et al., 2015). Despite considerable efforts, the vast majority of laboratory‐developed stroke therapies have failed to translate into clinically effective treatments (Albers et al., 2011; Saver, Jovin, Smith, & Albers, 2013). Current Food and Drug Administration (FDA)‐approved stroke therapies are limited to tissue plasminogen activator (tPA) and a limited number of stent‐retriever devices (Lu et al., 2014). These therapies are only effective in patients during the acute phase of injury and do not carry any regenerative potential for damaged tissues (Savitz et al., 2011).

Induced‐pluripotent‐stem‐cell‐derived neural progenitor cells (iNPCs) have recently garnered significant interest as a personalized regenerative cell therapy for stroke (Hyun, 2010; Savitz et al., 2011). iNPCs can potentially be derived from the patient's own body, thus eliminating the potential of rejection upon transplantation (Araki et al., 2013; Guha, Morgan, Mostoslavsky, Rodrigues, & Boyd, 2013). Several groups have already demonstrated beneficial effects of iNPCs in rodent models of ischemic stroke (Chang et al., 2013; Gomi et al., 2012; Jensen, Yan, Krishnaney‐Davison, Al Sawaf, & Zhang, 2013; Oki et al., 2012; Polentes et al., 2012; Tatarishvili, 2014; Tornero et al., 2013). iNPCs grafts implanted into rodents after middle cerebral artery occlusion (MCAO) were able to survive for up to 5 months with no evidence of tumorigenesis (Oki et al., 2012; Tornero et al., 2013). In addition, rodents receiving iNPCs following MCAO demonstrated improved functional recovery on various tests including the sticky tape/adhesive removal test, staircase test, rotarod test, and the modified neurologic severity score (mNSS) (Chang et al., 2013; Eckert et al., 2015; Gomi et al., 2012; Oki et al., 2012; Tornero et al., 2013). While iPSC‐derived therapies hold great promise in rodent stroke models, previous translational failures between rodents and humans indicate the need for further evaluation of this therapy before proceeding to clinical trials.

The repeated failures in translation between the laboratory and the clinical setting prompted the development of the stroke therapy academic and industry roundtable (STAIR) recommendations (Albers et al., 2011; Fisher et al., 2009; Roundtable, 1999; Saver et al., 2013). One of the recommendations included pre‐clinical testing of developed therapies in multiple animal models, preferably with inclusion of a gyrencephalic species (Roundtable, 1999). To address this need, our research group recently developed a gyrencephalic pig model of permanent right middle cerebral artery (MCA) ischemic stroke (Platt et al., 2014). Repeatable and reliable structural lesions in the pig model were demonstrated with both magnetic resonance imaging (MRI) and histology; however, functional outcome assessment was only limited to gait analysis (Duberstein et al., 2014). Another STAIR roundtable recommendation was to report the effect of proposed therapies on the acute and long‐term functional outcomes of tested animal models (Fisher et al., 2009). The failure of many stroke therapy clinical trials can partially be blamed on a lack of long‐term functional outcome evaluation in the preclinical animal model (Corbett & Nurse, 1998; Hunter, Mackay, & Rogers, 1998; Hunter et al., 2000; Roundtable, 1999). In humans, functional recovery can be thought of in terms of various neurologic domains: motor, sensory, vision, affection, cognition, and language (Kelly‐Hayes et al., 1998). Following a stroke, these domains are assessed using a variety of scales and indices including the modified Rankin Score (mRS), American Heart Association Stroke Outcome Classification Score (AHA.SOC), Barthel index (BI), NIH Stroke Scale (NIHSS), the Glasgow Outcome Scale (GOS), and the Canadian Neurologic Scale (CNS) (Heiss & Kidwell, 2014). The goal of these evaluations is to determine the degree of neurologic recovery and function through quantification of patient neurologic deficits, quality of life, and functional independence in activities of daily living (Heiss & Kidwell, 2014). The importance of these scales is most evident in the context of randomized clinical trials, most of which use the mRS or BI as the standard to dichotomize good from poor outcomes (Weisscher, Vermeulen, Roos, & de Haan, 2008).

The vast majority of ischemic strokes in humans involve the MCA, and several well‐established rodent models of MCAO exist (Bederson et al., 1986; Markgraf et al., 1992; Zausinger, Hungerhuber, Baethmann, Reulen, & Schmid‐Elsaesser, 2000). In MCAO, affected brain regions usually include large areas of the sensorimotor cortex and basal nuclei (Bederson et al., 1986; Modo et al., 2000; Ringelstein et al., 1992). Humans affected by MCAO can develop symptoms including, but not limited to, aphasia, hemiparesis, hemineglect, dysphagia, impaired cognition, and urinary/fecal incontinence (Banks & Marotta, 2007). Many of the criteria and scoring parameters assess language, emotion, and cognition; however, they do not translate well into non‐human species. As such, several animal‐specific scales and tests have been designed to assess similar representative outcomes in laboratory species (Corbett & Nurse, 1998; Hunter et al., 2000; Janssen et al., 2013). These tests have been designed to assess learning, memory, motor skills, and asymmetrical neurologic deficits (Modo et al., 2000).

Functional outcome assessments in rodent models of stroke are extremely variable and a gold standard does not exist (Corbett & Nurse, 1998; Hunter et al., 1998, 2000). Many studies employ a composite scoring system (Bederson Scale or mNSS) and/or perform a multitude of specific tests (e.g., Rotarod, Morris Water Maze) and grade each individually (Corbett & Nurse, 1998; Encarnacion et al., 2011; Hunter et al., 1998; Schaar, Brenneman, & Savitz, 2010). One of the earliest composite neurologic grading systems is the Bederson Scale (Bederson et al., 1986; Hunter et al., 1998). A large number of grading systems are based on this scale, which uses limb placement and circling as measurements of sensorimotor deficits (Bederson et al., 1986; Longa, Weinstein, Carlson, & Cummins, 1989; Schaar et al., 2010; Zausinger et al., 2000). Another popular composite score scale is the mNSS, which takes into account sensorimotor function, reflex, and balance tests (Schaar et al., 2010). However, there is currently no commonly performed composite scoring system for the pig stroke model.

In our recent study, MRI results demonstrated that iNPC‐treated stroked pigs had improved white matter integrity, brain metabolism, and cerebral blood volume in the ipsilateral stroked hemisphere and histological results showed improved neuron survival, increased neurogenesis, and decreased immune response relative non‐treated control stroked pigs (Baker et al., 2017). However, the question remained if these tissue and cellular level changes lead to functional recovery. Utilizing behavioral data collected from these same animals, the purpose of this study was to create a robust and repeatable post‐stroke assessment scale for stroke pigs. In this study, we demonstrated that this scale is able to consistently determine differences between non‐stroked and stroked animals with high repeatability within and between multiple assessors. In addition, we showed that iNPC treatment lead to improved recovery in postural reactions, body posture, mental status, and appetite in a pig ischemic stroke model.

## 2 MATERIALS AND METHODS

All procedures were conducted under guidelines approved by the University of Georgia Institutional Animal Care and Use Committee.

### 2.1 Animals

Eight male castrated 6‐month‐old Landrace pigs were used in the study. All pigs were obtained from the University of Georgia Swine unit and weighed between 65 and 80 kg at the start of the study and between 110 and 130 kg at the end of 12 weeks.

### 2.2 Permanent right MCAO

Pigs were sedated with an intramuscular injection containing xylazine (5 mg/kg), ketamine (5 mg/kg), midazolam (0.2 mg/kg), and butorphanol (0.2 mg/kg). All pigs were intubated with a cuffed endotracheal tube and maintained under gas anesthesia with administration of 1–2% isoflurane and oxygen. Mechanical ventilation was performed at a rate of 8–12 breaths/min using a tidal volume of 5–10 ml/kg throughout anesthesia. Intravenous fluids (Lactated Ringers Solution) were administered at a rate of 5–10 ml/kg throughout anesthesia. Further analgesia was provided with intramuscular injections of flunixin meglumine (Banamine^®^‐S; Merck Animal Health Cat# 01142) 2.2 mg/kg administered 30 min prior to surgery and every 24 hr, thereafter for 3 days postoperatively. Ceftiofur sodium (Naxcel^®^; Zoetis Cat# 009071) 4.4 mg/kg was administered intramuscularly at least 30 min prior to surgery. Doses were repeated every 24 hr for 3 days following surgery.

The surgical technique used to create a permanent right MCAO has been described in detail previously (Platt et al., 2014). Briefly, a right frontotemporal craniectomy and orbital rim ostectomy with partial zygomatic arch resection was performed on each animal. Bipolar cautery was used to permanently occlude the right MCA and some of its collateral branches near its origin. A 2 cm × 2 cm piece of porcine urinary bladder mucosa (ACell Vet™; Cat# 07‐834‐9561) was placed over the craniectomy defect prior to closure.

### 2.3 Magnetic resonance imaging

Twenty‐four hours following induction of right MCAO, each pig underwent MRI evaluation of the brain using a Siemens 16‐channel fixed‐site 1.5T MRI system. Pigs were sedated with xylazine (5 mg/kg), midazolam (0.2 mg/kg), and butorphanol (0.2 mg/kg) administered intramuscularly. All pigs were intubated with cuffed endotracheal tubes and maintained on 1–2% isoflurane throughout the MRI procedure. Peripheral intravenous catheters (18–22 g) were placed in the left or right auricular vein and in the left or right accessory cephalic veins for administration of Lactated Ringers Solution at a rate of 5–10 ml/hr during anesthesia.

T1‐weighted, T2‐weighted, T2 fluid attenuated inversion recovery (FLAIR), and diffusion‐weighted imaging (DWI) were performed in sagittal, transverse, and dorsal planes. Apparent diffusion coefficient (ADC) maps were generated from the DWI sequence.

### 2.4 Human‐induced pluripotent‐cell‐derived neural progenitor cells (iNPCs)

HIP™ human neural stem cells (ThermorFisher Scientific Cat# GSC‐4311, RRID:http://scicrunch.org/resolver/CVCL_RX99; hereafter “iNPC”) were expanded on growth factor reduced Matrigel™ (Corning™ Cat# 356231) diluted 1:100 with Neurobasal Medium (ThermoFisher Scientific Cat# 21103049) and kept under a passage number of 20. Daily media changes were performed using Neurobasal media (ThermoFisher Scientific) supplemented with 2% B‐27 Supplement (ThermoFisher Scientific Cat# 17504044), 1% non‐essential amino acids (ThermoFisher Scientific, catalog number 11140050), 2 mmol/L l‐glutamine (ThermoFisher Scientific Cat# 25030081), 1% penicillin/streptomycin (ThermoFisher Scientific Cat# 15140122), and 20 ng/ml basic fibroblast growth factor (bFGF) (R and D systems Cat# 233‐FG‐025). Upon confluence, cells were enzymatically suspended for passage using Accutase (Innovative Cell Technologies Cat# AT104) and replated at a density of 1:4.

### 2.5 Immunocytochemistry

iNPC were plated onto Matrigel‐coated four‐chambered glass slides for immunocytochemistry. Cells were fixed with 4% paraformaldehyde (Electron Microscopy Sciences) for 15 min and permeabilized with 0.1% Triton X‐100, 1% polyvinylpyrrolidone (PVP; Sigma‐Aldrich) in a 3% serum blocking solution. Cells were incubated with primary antibodies diluted in blocking solution for 1 hr at room temperature. Primary antibodies used were Nestin (Neuromics Cat# MO15012‐100, RRID:http://scicrunch.org/resolver/AB_2148919; dilution 1:200) and Sox1 (R and D Systems Cat# AF3369, RRID:http://scicrunch.org/resolver/AB_2239879; dilution 1:20). Secondary antibodies used were Donkey anti‐Goat IgG (H+L) Alexa Fluor 488 (ThermoFisher Scientific Cat# A‐11055, RRID:http://scicrunch.org/resolver/AB_2534102; dilution 1:1000), and Goat anti‐Mouse IgG (H+L) Alexa Fluor 594 (ThermoFisher Scientific Cat# A‐11032, RRID:http://scicrunch.org/resolver/AB_2534091; dilution 1:1000). Secondary antibodies were applied for 1 hr at room temperature to detect primary antibodies. Cells were washed and mounted with Prolong Gold with DAPI (ThermoFisher Scientific Cat# P36935) and imaged using SlideBook software version 4.1.0 (Intelligent Imaging innovations) on an Olympus IX‐81 microscope with Disc‐Spinning Unit (Olympus, Inc.).

### 2.6 Cell transplantation

For all eight animals that underwent MCAO, the surgical site was reopened 5 days post‐stroke for either a PBS‐only injection or an iNPC injection. Four pigs were assigned to each treatment group to receive either a PBS‐only (non‐treated) or an iNPC injection (iNPC‐treated). Pigs were anesthetized using the same protocol as described previously and the surgical site reopened via the same incision. Tissues were gently bluntly dissected to the level of the prior craniectomy and the previously placed porcine urinary bladder mucosa (ACell Vet™) was removed to expose the brain. For cell injections, the iNPCs were suspended in PBS at a concentration of 150,000 cells/μl. Two injections of 33.3 μl of the cell solution were administered through a glass syringe (Hamilton Co.) and 24‐g needle using a micro‐injector set to deliver the volume at a rate of 2 μl/min. Injections were administered at least 5 mm apart in the penumbra region of the stroke (as determined through 24‐hr post‐stroke MRI evaluation for each individual pig) at a depth of 6 mm from the surface of the brain at the junction of cortical gray and white matter. The needle was retracted at a rate of 1 mm/min following injection to prevent backflow of cells. For PBS‐only treatments, two injections of 33.3 μl of sterile PBS were injected in lieu of the cell solution in an identical manner. Following injections, a 2 cm × 2 cm piece of porcine urinary bladder mucosa (ACell Vet™) was placed over the craniectomy site prior to closure. All pigs were treated with flunixin meglumine (Banamine^®^‐S) 2.2 mg/kg administered 30 min prior to injection surgery and every 24 hr, thereafter for 3 days postoperatively. Ceftiofur sodium (Naxcel^®^) 4.4 mg/kg was administered intramuscularly at least 30 min prior to surgery with doses repeated every 24 hr for 3 days following surgery.

### 2.7 Post‐stroke assessment scale

A porcine post‐stroke neurologic assessment scale was created to include evaluation of individual parameters such as mentation, posture, gait, postural reactions, cranial nerves, appetite, and circling (Table 1). This scale was based on previously published post‐stroke clinical assessment scales in both pigs and dogs (Tanaka et al., 2008; Yamaguchi, Zhou, Heistad, Watanabe, & Zhang, 2004). Postural reactions were assessed by shifting the animal's weight over the center of balance for each individual limb through steady pressure applied by the assessor on the contralateral side of the animal. This was meant to mimic hopping tests performed in veterinary neurologic exams in dogs to assess conscious and unconscious proprioception. The maximum score associated with the highest degree of neurologic deficits was set at 30 and a normal neurologic exam score was set as zero.

**Table 1:** The post‐stroke assessment scale. Individual parameters were scored out of a range of 2–6. The highest possible score for animals most severely affected is 30 with normal or pre‐stroke animals scoring 0. More points were allotted to parameters that would have a more serious consequence for the pig such as appetite, gait, and mental status, whereas parameters such as head posture were allotted fewer points. Cranial nerves were given a higher allotment of points to incorporate the significance of brainstem deficits as assessed in coma‐scores in the acute stroke patient

Mental status	
Alert	0
Depressed/lethargic	1
Demented	2
Stuporous	3
Comatose	4
Appetite	
Eating well with no assistance (consumed all feed in 30 min)	0
Consumed more than 50% of food with no assistance in 30 min	1
Consumed 50% of food in 30 min with no assistance	2
Consumed less than 50% of food in 30 min with no assistance	3
Anorexic (not eating)	4
Head posture	
Erect/normal	0
Head raised on stimulation	1
Unable to raise head	2
Body posture	
Normal	0
Leaning to one side	1
Falling to one side	2
Extensor rigidity of limbs and alert (decerebellate)	3
Extensor rigidity of limbs and stuporous (decerebrate)	4
Circling	
No circling	0
Intermittent circling (note side)	1
Consistently circling (note side) or non‐ambulatory	2
Gait	
Normal all four limbs	0
Ambulatory with weakness of one limb (note limb)	1
Ambulatory with weakness/ataxia of both limbs on one side (note side)	2
Ambulatory with weakness/ataxia of all four limbs	3
Non ambulatory with intact motor movement of all limbs	4
Non ambulatory with paralysis of any of the limbs and intact nociception (note limbs)	5
Non ambulatory with paralysis of any of the limbs and loss of nociception (note limbs)	6
Postural reactions	
Hopping normal in all four limbs	0
Hopping slow in one limb (note limb)	1
Hopping slow in more than one limb (note limb(s))	2
Hopping absent in one or more limbs (note limb(s)) or non‐ambulatory	3
Cranial nerves	
Normal (no deficits)	0
Absent menace response unilaterally (note eye) with normal palpebral reflexes	1
Facial palsy with reduced palpebral reflexes and/or facial hypalgesia (note side)	2
Any of the above deficits with slow pupillary light reflexes and normal to reduced oculocephalic reflexes	3
Pinpoint pupils with reduced to absent oculocephalic reflexes ± pathologic/spontaneous nystagmus	4
Unilateral or bilateral unresponsive mydriasis with reduced to absent oculocephalic reflexes	5

Evaluations were performed at least 1–3 days prior to induction of stroke and repeated 1 day, 3 days, and 5 days post‐stroke as well as 1 day, 3 days, 1 week, 2 weeks, 4 weeks, 6 weeks, 9 weeks, and 12 weeks post‐injection. All examinations were physically performed by one individual (VL) and filmed with a digital camera (Canon Powershot D10). Filmed examinations were later viewed and scored by a non‐blinded observer who was aware of the time points at which pigs were evaluated. All filmed examinations were viewed and scored again by the same non‐blinded observer at a later time. Filmed exams were then relabeled and placed in random order for evaluation by an observer blinded to the treatment group and time points of each examination and to the first observer's scores. Only results from the scoring of the blinded observer were used in the final analysis to determine significance between iNPC‐treated and non‐treated pigs.

### 2.8 Statistics

All statistical analysis was performed using sas version 9.3 (SAS Institute, Inc). A two‐way ANOVA and a Tukey's post‐hoc *t* test were used to compare results between treatment groups and between the first and second assessments of the non‐blinded observer and the blinded observer. A *p* value <.05 was used to determine significance between groups.

## 3 RESULTS

### 3.1 iNPCs homogenously express the neural progenitor markers SOX1 and Nestin

**Figure 1:**
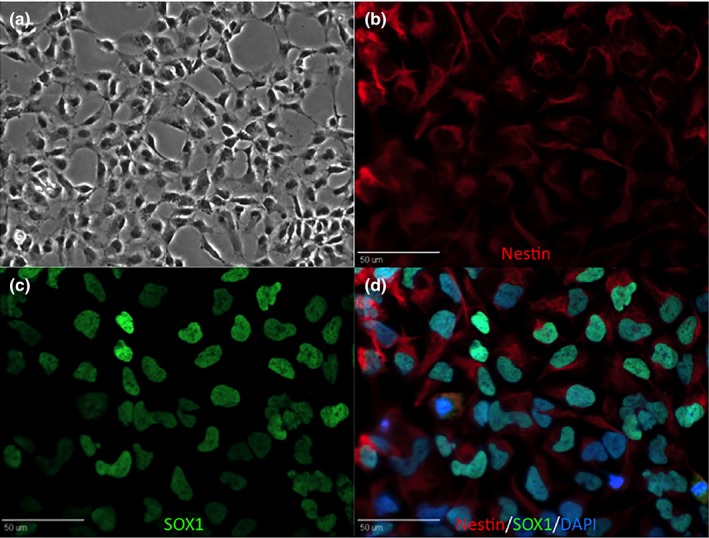
iNPCs show typical neural progenitor cell morphology on phase contrast at 20× magnification (a). Immunocytochemistry demonstrates positive expression of neural stem cell markers Nestin (b) and SOX1 (c). Merged image with DAPI (d)

iNPCs were observed to have a normal monolayer growth pattern with individual cells showing large cell bodies and extended processes consistent with neural progenitor cell morphology (Figure 1a). Immunocytochemistry results showed iNPCs co‐expressed the neural progenitor markers SOX1 and Nestin (Figure 1b–d). Greater than 95% of iNPCs were positive for SOX1 and Nestin.

### 3.2 MRI 24‐hr post‐surgery reveals ischemic stroke in the middle cerebral artery territory

**Figure 2:**
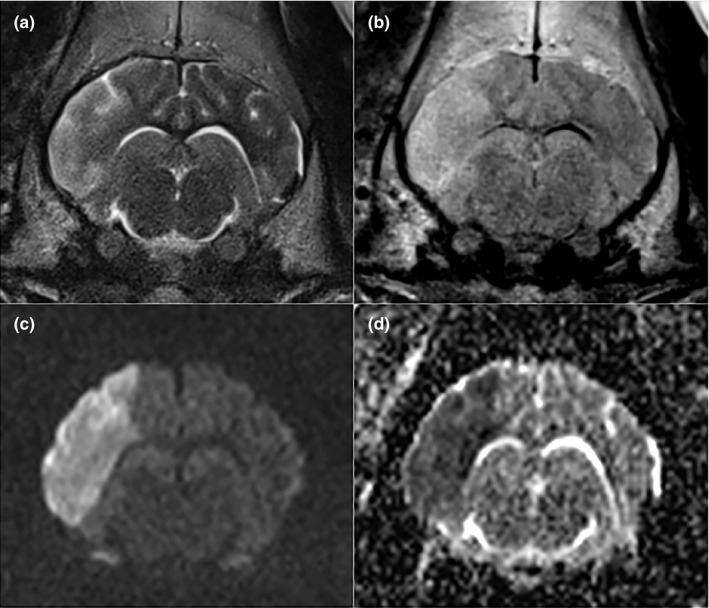
MRI performed 24 hr following MCAO demonstrates ischemic stroke in the territory of the middle cerebral artery. The affected area is hyperintense on T2‐weighted imaging (a) and T2 FLAIR (b) relative to normal grey matter. The region is hyperintense on DWI (c) with corresponding hypointensity on the ADC map (d) confirming cytotoxic edema

Twenty‐four hours following bipolar cauterization of the right MCA, all eight animals underwent MRI evaluation of the brain. An area of increased signal intensity was noted in the distribution of the right MCA in each pig on T2‐weighted (Figure 2a), T2‐FLAIR (Figure 2b), and DWI sequences (Figure 2c). A corresponding region of decreased signal intensity on ADC maps consistent with cytotoxic edema confirming ischemic infarction was identified in each animal (Figure 2d).

### 3.3 The post‐stroke assessment scale reliably detects functional changes in pigs after MCAO stroke and iNPC treatment

**Figure 3:**
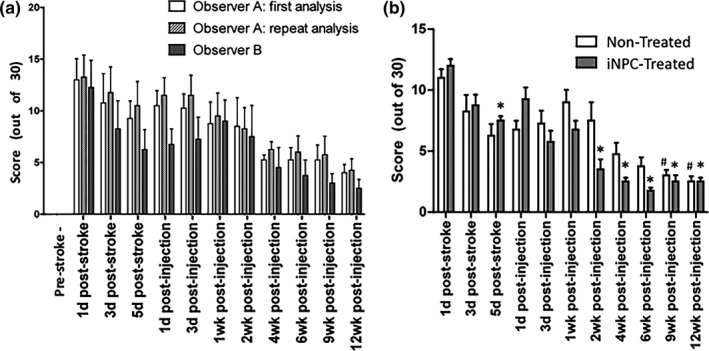
All post‐stroke time points for both treatment groups were significantly different from pre‐stroke scores (a). No significant differences were noted between observers or between different assessments by the same observer at any time point in pigs following MCAO (a). iNPC‐treated animals showed significant improvement (*) from 1 day post‐stroke scores by 2 weeks following iNPC‐injection (*p* < .05), whereas non‐treated animals did not reach this improvement level until 9 weeks post‐injection (#) (b)

Overall scores obtained through the post‐stroke assessment scale were able to demonstrate significant (*p* < .05) differences between pre‐stroke and post‐stroke animals at every time point for both observer A (non‐blinded) and observer B (blinded) at all time points (Figure 3a). No detectable differences were noted between initial and repeat assessments performed by the non‐blinded observer (*p* < .05). Likewise, no significant differences were detected between the overall scores assigned by the blinded observer and the non‐blinded observer at any time point (*p* < .05). This indicates that the assessment scale is reliable both between different observers and between assessments performed by one observer. Furthermore, iNPC‐treated pigs showed significant improvement in their overall total post‐stroke score relative to 1 day post‐stroke by 2 weeks post‐injection (Figure 3b). Non‐treated pigs did not demonstrate significant improvement until 9 weeks post‐injection.

### 3.4 iNPC treatment hastened recovery of postural reactions, posture, mental status, and appetite following MCAO

**Figure 4:**
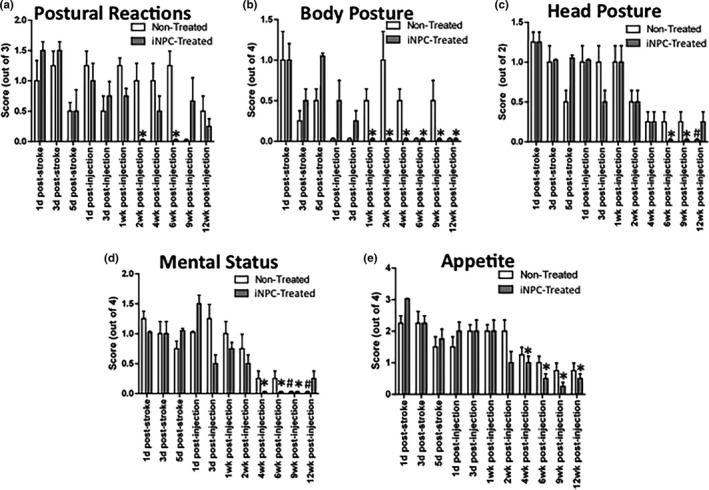
Significant improvement in postural reaction scores were noted in the iNPC treated group by 2 and 6 weeks post‐injection (a), whereas non‐treated pigs did not exhibit any improvement over 12 weeks. The body posture scores appeared to improve by 1 week post‐injection in iNPC‐treated pigs (b) with no improvement ever noted in the non‐treated pigs. Significant improvements in head posture scores were noted by 6 weeks post‐injection in iNPC‐treated pigs but not until 12 weeks post‐injection in non‐treated pigs (c). Improvements in mental status scores were noted by 4 weeks post‐injection in iNPC‐treated pigs, whereas non‐treated pigs did not show improvement until 9 weeks post‐injection (d). Appetite scores of iNPC‐treated pigs improved by 4 weeks post‐injection while non‐treated pigs did not show significantly improved appetites throughout the 12‐week period (e). * represent time points in the iNPC‐treated group where scores were significantly different from 1‐day post‐stroke scores (*p* < .05). *In the body posture graph (b) are an exception in that these were time points significantly different from 5 days post‐stroke (*p* < .05). # represents time points where the non‐treated scores were significantly improved from 1 day post‐stroke scores

Overall, iNPC‐treated MCAO stroke pigs demonstrated a faster functional recovery relative to non‐treated control pigs; however, unique parameters showed varying outcomes (Figure 4). An improvement in postural reactions was noted in iNPC‐treated pigs in the score between 1 day post‐stroke and scores at 2 and 6 weeks post‐injection, whereas non‐treated pigs did not exhibit improvement in their postural reaction scores over the 12‐week testing period (Figure 4a). iNPC‐treated pigs also demonstrated a significant improvement in their body posture scores by 1 week post‐injection compared to 5 days post‐stroke, whereas non‐treated pigs did not show any significant improvements in their body posture scores (Figure 4b). A similar trend was observed in head posture scores; iNPC‐treated pigs exhibited improvement in head posture 6 and 9 weeks post‐injection compared to 1 day post‐stroke, whereas non‐treated pigs did not show an improvement until 12 weeks post‐injection (Figure 4c). iNPC‐treated pigs also showed more rapid recovery of their mental status scores with significant improvements by 4 weeks post‐injection compared to 1 day post‐stroke while this improvement was not observed in the non‐treated group until 9 weeks post‐injection (Figure 4d). There was also a significantly improved appetite score in the iNPC‐treated pigs by 4 weeks post‐injection relative to 1 day post‐stroke while no significant improvement in appetite was ever noted in control pigs over 12 weeks (Figure 4e).

### 3.5 iNPC treatment does not improve rate of recovery of circling tendency, cranial nerve function, or gait

**Figure 5:**
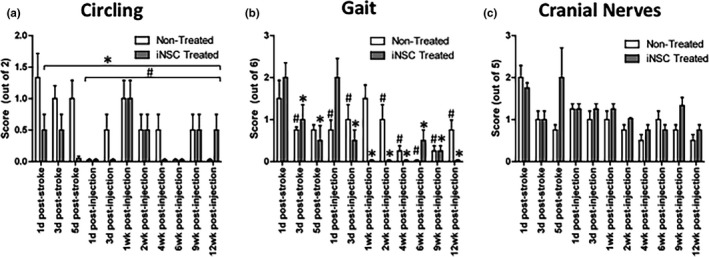
Spontaneous recovery was noted in circling and gait scores within a few days post‐MCAO (a and b). * represents time points where scores recovered to pre‐stroke levels in iNPC‐treated pigs. # represents time points where scores recovered to pre‐stroke levels in non‐treated pigs

For circling and gait, both treatment groups did not exhibit any significant improvement by 12 weeks post‐injection compared to their 1 day post‐stroke scores (Figure 5a,b). However, the post‐stroke scores for both parameters were only mildly elevated, and spontaneous recovery to pre‐stroke scores for gait and circling was noted in both treatment groups by 1–5 days post‐stroke. No significant difference was noted between treatment groups at any time point for these parameters. Cranial nerve function scores were significantly different from pre‐stroke scores at all time points with no evidence of recovery from 1 day post‐stroke scores over 12 weeks in either treatment group (Figure 5c).

## 4 DISCUSSION

In this study, we have developed a porcine post‐stroke functional outcome assessment scale that was sensitive enough to detect changes between normal and stroked animals, and between iNPC‐treated and non‐treated pigs with high intra‐ and inter‐observer repeatability. Furthermore, we utilize this assessment scale to show that human iNPC therapy leads to significant improvement in functional neurologic outcome across multiple parameters in a pig ischemic stroke model. Scores for the individual parameters showed more variability between observers; however, this did not affect repeatability of the overall scale score. This supports the use of the overall score of this scale in future studies to assess the effect of iNPC therapy or other novel therapies on neurologic function in pigs following stroke.

We recently demonstrated that iNPC treatment leads to tissue and cellular level recovery in stroked pigs; however, a robust and repeatable post‐stroke assessment scale was needed to demonstrate functional recovery (Baker et al., 2017). The developed pig stroke assessment scale showed that animals that received iNPC‐injections demonstrated significantly faster recovery of postural reactions, body posture, head posture, mental status, and appetite relative to non‐treated animals. Body posture, head posture, and postural reactions are a reflection of sensorimotor status. The faster rates of recovery noted here are similar to findings in rodent studies of neural progenitor cell therapy following stroke (Chang et al., 2013; Eckert et al., 2015; Gomi et al., 2012). Head and body posture scores were designed to broadly assess the sense of balance as well as unconscious and conscious proprioception similar to the beam walk and rotarod tests used in rodents. The improvements in these parameters are similar to improvements of the aforementioned tests noted in rodents following iNPC therapy as shown by Eckert et al. (2015) and Chang et al. (2013). Postural reaction assessments in the pigs in this study were scored similarly to the sensory test portion of the rodent mNSS and were designed to test unconscious and conscious proprioception. Rodent mNSS scores were previously demonstrated to improve more rapidly following iNPC injections by Gomi et al. (2012) and Chang et al. (2013). In contrast to the rodent studies, however, the rapid recovery in the iNPC‐treated pigs did not result in significant differences in neurologic scores between iNPC‐treated and non‐treated groups by the endpoint of the study (12 weeks post‐stroke) due to spontaneous recovery of the non‐treated pigs. This difference may be due to the much shorter follow‐up times in rodent studies with endpoints being 9 weeks or fewer, thus allowing less time for spontaneous recovery to occur in the chronic stage post‐stroke. An exception to this is the study by Polentes et al. in which rodents were followed for 4 months following stroke and cell transplantation (Polentes et al., 2012). In their study, animals receiving cell grafts demonstrated sustained improvements over vehicle‐injected animals for tape‐removal and apomorphin‐induced rotation behavioral tests. However, both grafted and non‐grafted animals demonstrated spontaneous recovery simultaneously on assessments of the Montaya stair case test and the mNSS within 1 month post‐stroke (Polentes et al., 2012). Scores for individual parameters within the stroke scale did not retain significant differences from pre‐stroke scores throughout the 12‐week test period indicating spontaneous recovery in both treatment groups. This is similar to spontaneous recovery rates in humans which has been reported to occur by about 10 weeks post‐stroke; albeit the degree of recovery in humans is significantly less (Kwakkel & Kollen, 2013).

In addition to faster sensorimotor recovery, we show that iNPC‐treated pigs also demonstrated faster recovery of appetite over control animals. Human post‐stroke outcome assessment scales such as the BI and Functional Independence Measure (FIM) account for activities of daily living such as feeding and urinary/fecal continence (Dromerick, Edwards, & Diringer, 2003; Kwakkel & Kollen, 2013). It has been proposed that scales incorporating activities of daily living, such as feeding, are more sensitive to the level of disability and recovery following ischemic stroke than scales like the Modified Rankin Score (MRS) (Dromerick et al., 2003). If tasks like feeding are more sensitive, the rapid improvement of appetite noted in the iNPC‐treated pigs and lack of improvement in the control pigs may be the most compelling indicator that iNPC therapy improved recovery in pigs following MCAO.

Cranial nerve function did not exhibit significant improvement in either treatment group over the 12‐week testing period. The most common cranial nerve deficits were contralateral menace response and facial hypalgesia, which are consistent with injury to the sensorimotor cortex. The lack of significant improvement may be due to the grading scheme for cranial nerve deficits where higher scores were more reflective of midbrain and medullary dysfunction, which are areas that are not injured by MCAO. Future cranial nerve scores may be more sensitive if more weight is placed on menace response and facial hypalgesia rather than testing cranial nerves that originate in the midbrain or medulla.

Both treatment groups showed spontaneous improvement in their gait and circling scores within a few days post‐stroke before iNPC treatment occurred. The spontaneous recovery of gait scores by 3 days post‐MCAO in this study may be a reflection of the predominant role of extrapyramidal brainstem centers in gait generation in pigs rather than corticospinal tracts (King, 1987). In addition, the method of gait assessment used in this study (gross visual observation) may not have been as sensitive to dysfunction as computerized gait analysis performed with limb stride or step height measurements (Duberstein et al., 2014). More specialized gait analyses, however, require special equipment and setup, which would make the post‐stroke assessment scale less user‐friendly and globally transferable. Circling was placed in the scale as a crude measurement of cognitive dysfunction and hemi‐inattention and was graded on a scale from 0 to 2, making it one of the smallest contributors to the overall score. The narrow grading scale for this parameter may have altered its sensitivity and accuracy. Specifically, this test may not have been sensitive if the pigs were not observed for prolonged periods of walking. An induced‐rotation test, such as the apomorphine test used in rodents, may need to be developed to detect more subtle differences between treatment groups (Li et al., 2004; Schaar et al., 2010; Vorhees & Williams, 2006). The development of such tests for pigs following MCAO warrants further investigation.

The faster recovery noted in the overall score of iNPC‐treated pigs could be a reflection of the anti‐inflammatory and trophic factors secreted by iNPCs. The rapid onset of improvement in the cell‐treated group and lack of significant difference between treatment groups at 12 weeks post‐injection would argue against neuronal regeneration being the major mechanism of action. iNPCs have previously been reported to reduce inflammatory cytokines such as TNF‐α, IL‐6, and IL‐1β, in addition to reducing microglial activation and mitigating neuronal loss which is correlated to improved neurologic outcome (Eckert et al., 2015; Oki et al., 2012; Polentes et al., 2012; Tatarishvili, 2014).

Given the spontaneous recovery seen in gait and circling, these parameters may be excluded from future post‐stroke functional outcome assessment scales. Alternatively, more sensitive testing for these parameters, possibly through development of a porcine apomorphine‐induced rotation test and/or computerized and measured gait analyses, may allow for better detection of disability and differences between treatment groups in future studies. The disparity between appetite scores of the iNPC‐treated and non‐treated pigs may indicate that this is the most sensitive parameter in the functional outcome scale (Dromerick et al., 2003). In addition, appetite may also be the most translatable to human functional outcome scales, and future modifications to the porcine scale should be weighted accordingly.

The post‐stroke assessment scale designed in this study offers a robust and repeatable means of evaluating functional outcomes in a large animal model of stroke. This will be of significant value to the field as future studies focus on the pig as a translational animal model for neural disease and injury. In addition, we demonstrated for the first time that iNPC therapy shortened functional recovery time in a large animal stroke model. Shorter recovery times could have significant effects on human quality of life and the cost associated with post‐stroke hospitalization and long‐term care.
